# Molecular Mechanisms of Healthy Aging: The Role of Caloric Restriction, Intermittent Fasting, Mediterranean Diet, and Ketogenic Diet—A Scoping Review

**DOI:** 10.3390/nu16172878

**Published:** 2024-08-28

**Authors:** Roxana Surugiu, Mihaela Adela Iancu, Ștefănița Bianca Vintilescu, Mioara Desdemona Stepan, Daiana Burdusel, Amelia Valentina Genunche-Dumitrescu, Carmen-Adriana Dogaru, Gheorghe Gindrovel Dumitra

**Affiliations:** 1 Department of Biochemistry, University of Medicine and Pharmacy of Craiova, St. Petru Rares, No. 2-4, 200433 Craiova, Romania; roxana.surugiu@umfcv.ro (R.S.); daiana.burdusel@gmail.com (D.B.); 2 Department of Internal Medicine (Cardiology, Gastroenterology, Hepatology, Rheumatology, Geriatrics), Family Medicine, Labor Medicine, “Carol Davila” University of Medicine and Pharmacy, 050474 Bucharest, Romania; adela.iancu@umfcd.ro; 3 Department of Infant Care-Pediatrics-Neonatology, University of Medicine and Pharmacy of Craiova, St. Petru Rares, No. 2-4, 200433 Craiova, Romania; vintilescubianca92@gmail.com (Ș.B.V.); desdemona.stepan@umfcv.ro (M.D.S.); 4 Department of Internal Medicine, University of Medicine and Pharmacy of Craiova, St. Petru Rares, No. 2-4, 200433 Craiova, Romania; 5 Department of Family Medicine, University of Medicine and Pharmacy of Craiova, St. Petru Rareș, No. 2-4, 200349 Craiova, Romania; gindrovel.dumitra@umfcv.ro

**Keywords:** dietary interventions, caloric restriction, intermittent fasting, mediterranean diet, ketogenic diet, healthy aging, longevity

## Abstract

As the population ages, promoting healthy aging through targeted interventions becomes increasingly crucial. Growing evidence suggests that dietary interventions can significantly impact this process by modulating fundamental molecular pathways. This review focuses on the potential of targeted dietary strategies in promoting healthy aging and the mechanisms by which specific nutrients and dietary patterns influence key pathways involved in cellular repair, inflammation, and metabolic regulation. Caloric restriction, intermittent fasting, the Mediterranean diet, as well as the ketogenic diet showed promising effects on promoting healthy aging, possibly by modulating mTORC1 AMPK, an insulin signaling pathway. By understanding the intricate interplay between diet and molecular pathways, we can develop personalized dietary strategies that not only prevent age-related diseases, but also promote overall health and well-being throughout the aging process.

## 1. Introduction

The global population has exhibited remarkable growth over the past century, nearly tripling from approximately 3 billion individuals in 1950 to a staggering 8 billion in 2022, according to data from the United Nations, Department of Economic and Social Affairs, Population Division [1]. This surge in population has occurred in tandem with a notable increase in life expectancy, which has risen from 45.51 years in 1950 to 73.16 years in 2023. Projections indicate a further upward trajectory, with an anticipated average life expectancy of 81.88 years by the year 2100 [1].

However, this demographic shift is accompanied by significant aging of the global population, attributed to declining fertility rates and prolonged life expectancies [2]. Although life expectancy stands as a widely utilized gauge of a population’s overall health, its interpretation is not without complexities influenced by biases and evolving trends [3]. As societies struggle with unprecedented aging dynamics, a comprehensive understanding of these interconnected factors becomes essential for informed policymaking and societal adaptation. 

Over the past 30 years, global health has improved with declining age-standardized DALY (disability-adjusted life year) rates. However, due to aging and population growth, total DALYs have held steady, with a notable shift that reveals more DALYs among older individuals [4]. This emphasizes the importance of understanding how aging will affect future health needs, linked to population transitions and health dynamics. The widely prevalent parameter for assessing aging functionality is chronological age (CA). Nevertheless, the progression of the aging process transpires gradually. Aging is a remarkably personalized phenomenon characterized by substantial inter-individual and intra-individual variabilities [5]. Due to the substantial variability observed among individuals, the use of chronological age (CA) as a sole indicator does not accurately capture the physiological changes associated with the aging process [6]. Researchers have attempted to find an alternative biological indicator to measure age because CA is not an accurate indicator of biological aging [7]. The assessment of biological aging employs indices to quantify the extent of aging and to distinguish between individuals with the same CA but different biological activities [8].

Since the extension of lifespan has not been paralleled by a corresponding increase in healthspan, it poses a significant challenge for our societies. Addressing this challenge requires the critical task of identifying key factors that can impact health during advanced age. Firstly, the definition of the term is required. The term “healthspan” refers to the amount of time an individual spends in an overall condition of well-being and functional capacity, and is dependent on the definition of “health”. In 1948, the World Health Organization defined health as being “a state of complete physical, mental, and social well-being, and not merely the absence of disease or infirmity” [9], but more recently Rozanski discussed the importance of vitality in the concept of well-being [10]. Furthermore, it is imperative to develop and validate interventions aimed at retarding or mitigating the aging process and its related health conditions. The process of aging is intricately linked to the presence of comorbidities and age-related diseases; thus, it is imperative to utilize relevant preclinical models involving aged animals, both with and without comorbidities [11], as well as to take into consideration lifestyle changes [12]. For promoting healthy aging, nutritional interventions have emerged as a highly promising avenue. A growing body of evidence indicates that these interventions can have a measurable effect on the health outcomes of individuals [13,14,15,16,17].

## 2. Molecular Mechanisms and Dietary Influences in Aging

Over the years, extensive research has focused on the molecular mechanisms underlying aging, revealing intricate networks of cellular pathways that influence the rate of aging and lifespan. Among these mechanisms, a significant focus has been on the activity of serine/threonine kinase mTORC1, belonging to the phosphoinositide 3-kinase-related kinase family (PIKK) [18], which has been demonstrated to correlate with extended lifespan in various genetic models with reduced mTORC1 signaling. mTOR forms two distinct protein complexes known as mTORC1 and mTORC2, with unique protein subunits responsible for phosphorylating different substrates [19]. mTORC1 governs pathways critical for proliferation, survival, and autophagy, whereas mTORC2 is involved in actin organization and metabolic regulation, particularly in insulin signaling [20]. Furthermore, mTOR signaling exhibits continuous activity during senescence, stemming from factors such as replicative depletion, oncogenic stimuli, and various stressors [21]. This sustained activation may contribute to geroconversion, the transition from proliferation to senescence, characterized by the absence of growth inhibition [22,23].

Among the various factors influencing aging, diet has emerged as a critical determinant of healthspan and longevity. Dietary restriction (DR), the reduction of calorie intake without malnutrition [24], has long been recognized for its remarkable ability to extend lifespan and delay the onset of age-related pathologies across a wide range of species, from yeast to mammals [25]. The profound effects of DR on aging have spurred an intense investigation into its molecular mechanisms and physiological consequences. DR exerts its anti-aging effects through multiple interconnected pathways, including the modulation of oxidative stress [26], inflammation [27], gut microbiota [28], and cellular senescence [29]. Central to the regulation of aging and lifespan is the target of the rapamycin (TOR) signaling pathway [30], a highly conserved nutrient-sensing pathway that integrates environmental cues to orchestrate cellular responses related to growth [31], metabolism [32,33], and various cellular processes—including protein synthesis [34], autophagy [35], and mitochondrial function [36]—in response to nutrient availability and energy status. 

Intermittent fasting can influence energy signaling pathways mediated by cAMP-responsive element binding protein (CREB) and AMP-activated protein kinase (AMPK), as well as the pro-growth mTOR pathways and the expression of circadian clock genes [37]. Fasting-sensitive AMPK is activated by increased AMP levels and, in turn, can alter the circadian clock by controlling critical circadian clock regulators [38]. Furthermore, the Mediterranean diet, which is rich in omega-3 fatty acids, has been associated with significant anti-inflammatory effects. These effects are believed to occur through the binding of omega-3 fatty acids to the G-protein–coupled receptor 120 (GPR120) and subsequent inhibition of NLRP3 inflammasome activity [39,40]. 

The ketogenic diet (KD) exhibits neuroprotective effects in the central nervous system (CNS) by suppressing glycolysis and promoting the production of ketone bodies (KBs) [41]. Inhibition of glycolysis, a fundamental aspect of dietary restriction as well, contributes to the regulation of insulin secretion, enhancing insulin sensitivity and improving glucose tolerance, ultimately promoting improved mitochondrial function and autophagy [42,43]. These metabolic effects are associated with delayed onset of age-related conditions and extended lifespan across various species [44]. 

**Figure 1:**
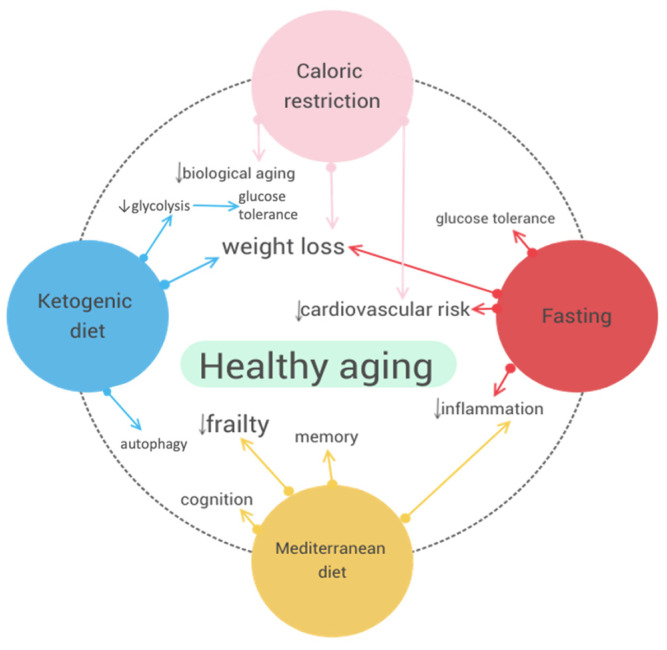
Main effects of dietary interventions on healthy aging.

To visually summarize the key mechanisms and benefits of each dietary intervention discussed, Figure 1 illustrates the main effects of caloric restriction, intermittent fasting, the Mediterranean diet, and the ketogenic diet on promoting healthy aging (Figure 1).

The aim of this review is to explore the potential of dietary interventions in promoting healthy aging by examining their effects on molecular pathways associated with cellular repair, inflammation, and metabolic regulation. Specifically, this review focuses on caloric restriction, intermittent fasting, the Mediterranean diet, and the ketogenic diet—each of which has been extensively studied for its unique mechanisms in modulating key pathways like mTORC1, AMPK, and insulin signaling, which are implicated in longevity and the prevention of age-related diseases. These diets were selected for their prominence in current research and their varied approaches to promoting health and longevity. By understanding these mechanisms, the review seeks to inform the development of personalized dietary strategies that enhance healthspan and overall well-being throughout the aging process. 

## 3. Methodology

To conduct a detailed examination of the impact of various diets and their molecular pathways associated with aging, this literature review employs an in-depth analysis of published research available in databases such as PubMed, Scopus, and Web of Science. The search strategy includes a combination of specific keywords and their synonyms, using Boolean operators to refine the results: (“dietary interventions” OR “dietary practices” OR “nutritional strategies”) AND (“molecular pathways” OR “cellular mechanisms”) AND (“aging” OR “senescence”) AND (“longevity” OR “lifespan”). Both primary research articles and review papers published between 2000–2024, in English are included to ensure a comprehensive overview. This review specifically focuses on caloric restriction, intermittent fasting, the Mediterranean diet, and the ketogenic diet, selected for their prominence in current research and varied approaches to promoting health and longevity. Exclusion criteria include studies published in languages other than English to ensure consistent data interpretation, as well as research published prior to 2000, unless deemed foundational or landmark studies.

## 4. Dietary Interventions

### 4.1. Caloric Restriction

Caloric restriction (CR), which is characterized by a sustained reduction in overall caloric intake while assuring adequate nutrition, is the most effective non-genetic intervention known to date for extending both lifespan and healthspan [24,45]. This intervention has garnered significant attention and has been extensively researched in a wide range of species. It has been shown to extend the lifespan of budding yeast (Saccharomyces cerevisiae) [46], enhance various physiological aspects associated with aging in nematode worms (Caenorhabditis elegans) [47,48] and fruit flies (Drosophila melanogaster) [49,50], and demonstrate promising results in improving overall health during aging. In rodents, it has been found to lead to extended lifespan, reduced pathological conditions, and improved metabolic well-being [51,52,53], as well as improved behavioral response post-stroke [54]. Additionally, this intervention has shown potential in enhancing metabolic health and preventing obesity, as well as delaying the onset of age-related conditions such as sarcopenia, presbycusis, and brain atrophy in Rhesus monkeys [55,56,57]. 

In a study conducted by Ravussin et al. [58], a two-year intervention involving 218 non-obese individuals aged 21 to 51 was undertaken. The participants were randomly assigned to either a 25% calorie restriction group or an ad libitum diet group, with a BMI range of 22.0 to <28 kg/m^2^. The study aimed to assess the effects of these interventions on aging indicators. The results indicated notable outcomes, including weight loss, reduced levels of circulating tumor necrosis factor-α (TNF-α), improvements in lipid profiles, resting metabolic rate (RMR), and total daily energy expenditure (TDEE). The intervention demonstrated its efficacy in promoting weight loss and mitigating cardiometabolic risk factors. However, the long-term implications of these effects on aging and healthspan remain uncertain [58]. A follow-up study using data from the CALERIE trial demonstrated that caloric restriction resulted in a significant reduction in the pace of biological aging in contrast to the ad libitum diet, by using the Klemera–Doubal method (KDM) for calculating human biological age. Ad libitum participants displayed an annual biological age increase of 0.71 years, whereas those practicing caloric restriction exhibited a slower increase of 0.11 years per year. This highlights a considerable deceleration of aging within the caloric restriction group [59].

### 4.2. Intermittent Fasting

Intermittent fasting (IF) diets involve eating patterns where individuals undergo extended periods (e.g., 16–48 h) with minimal or no energy intake, followed by regular food consumption periods, in a repeated manner [60]. The intermittent fasting regimen demonstrated a successful extension of lifespan in C. elegans [61,62] and Drosophila melanogaster [63], but in rodents, the results remain inconclusive [64,65].

In a recent study, 60 healthy individuals (with no history of fasting) aged 48 to 52 years with a BMI between 25.37 and 25.66 were divided into two groups: 30 engaged in alternate-day fasting (ADF) for 4 weeks, the rest continued ad libitum diet. Another group of 30 individuals was enrolled from a cohort of people that followed ADF for more than six months. ADF consisted of strict 36 h periods without caloric ingestion (fast days) followed by 12 h intervals of ad libitum food consumption (feast days), resulting in a calorie deficit of 37.40% in the ADF group on average. Notably, the ADF demonstrated improvements in body composition, as evidenced by a decrease in body mass index (BMI) of more than 1 kg/m^2^. Indicators of cardiovascular health, including systolic and diastolic blood pressure, heart rate, arterial and pulse pressure, and pulse wave velocity, were also improved. According to the Framingham risk score, the short-term ADF regimen also substantially reduced the risk of future adverse cardiovascular events by approximately 1.42 percent. Long-term ADF administration resulted in benefits such as reduced cardiovascular risk, improved cholesterol levels, and thyroid function modulation, with no adverse effects. In addition, long-term ADF was associated with increased secretion of parathyroid hormone (PTH), decreased levels of sICAM-1 (a biomarker associated with age-related diseases and inflammation), and depletion of the pro-aging amino acid methionine [66].

Twenty-four healthy men and women aged 55 to 79 participated in a study exploring time-restricted feeding (TRF), where participants fasted for 36 h periods and then had 12 h ad libitum eating intervals, divided into 2 groups, over six weeks. The study aimed to assess the effects of TRF on various health parameters. Participants successfully reduced their caloric intake window by approximately 4 h per day, resulting in a 33% reduction in their daily feeding period. TRF did not negatively affect diet composition or daily energy intake, and participants maintained their body mass. Hunger sensations were reduced during TRF, and TRF led to a small reduction in heart rate during light and moderate exercise. However, TRF did not influence cardiovascular function, resting blood pressure, or oxidative stress and inflammation markers. The study demonstrated that TRF is feasible and may have some positive effects on hunger and heart rate, but did not significantly impact other measured parameters [67].

### 4.3. Mediterranean Diet

The Mediterranean diet is known as being low in saturated fat and high in vegetable oils, primarily observed in Greece and Southern Italy [68], and is the most documented diet for slowing the development of age-associated conditions [69]. The Mediterranean diet has consistently demonstrated a protective effect against risk factors associated with age-related diseases across numerous studies [70,71,72]. This dietary pattern is distinguished by its emphasis on abundant consumption of unrefined cereals, fruits, vegetables, legumes, and olive oil. It also includes a moderate intake of dairy products and alcohol, while maintaining low consumption of meat, a synergistic blend of vital minerals, antioxidants, and compounds with anti-inflammatory properties that might have the potential to elicit hormetic effects [73,74]. Without any doubt, the Mediterranean diet is widely recognized as the primary dietary strategy for attaining an ideal combination of vital nutrients, antioxidants, and other beneficial substances that actively promote the progression of a healthy aging process [14].

In a study conducted by Varela–Lopez et al. [75], 72 male Wistar rats were examined to determine the effects of lifelong consumption of various fat sources abundant in monounsaturated (virgin olive oil), n6 polyunsaturated (sunflower oil), or n3 polyunsaturated (fish oil) fatty acids on the liver function of aged rats. The results indicated that rodents consuming virgin olive oil had lower protein carbonyl levels compared to those consuming sunflower and fish oils. Notably, aged rats in the virgin olive oil group had increased expression of eight genes, including two genes associated with mitochondrial dysfunction (Atp5d and Ndufa9), four genes associated with oxidative stress (Keap1, Map2k6, Pik3c2g, and Rras), and two genes associated with telomere length (Xrcc6 and Xrcc1) [75].

In the study conducted by Gensous et al. [76], 1279 participants aged 65 to 79, without significant chronic diseases were arbitrarily divided into intervention and control groups, stratified by age, gender, frailty status, and BMI. The purpose of the study was to evaluate the effects of a Mediterranean-style diet for one year on several parameters, including BMI, DNA methylation age, and epigenetic age acceleration. The results demonstrated that both Italian and Polish participants increased their adherence to the Mediterranean diet significantly. In both the Italian and Polish cohorts, there was a significant relationship between baseline epigenetic age acceleration measures and those observed after a 12-month nutritional intervention. Remarkably, the intervention involving a diet similar to the Mediterranean led to a statistically significant rejuvenation effect in the Polish subjects, as measured by AgeAccel (−1.47 years of AgeAccelDiff and of −1.36 years of IEAADiff)—intrinsic epigenetic age acceleration, in the subgroup of Polish females [76].

Another study that involved 1279 relatively healthy older adults aged 65–79 years from five European centers divided them into two groups: control, following a habitual diet, and “intervention”, which received personalized dietary advice based on Mediterranean-like principles tailored to the dietary needs of older adults from the included countries (NU-AGE). The study aimed to investigate the effects of NU-AGE’s dietary intervention on age-related cognitive decline over 1 year. The results showed that higher adherence to the NU-AGE diet was associated with significant improvements in global cognition (β = 0.20, *p* = 0.046) and episodic memory (β = 0.15, *p* = 0.025) compared to individuals with lower adherence. The study suggests that the NU-AGE diet may have a positive impact on cognitive function in older adults [77].

The follow-up study within the NU-AGE project involved 612 participants with the primary aim being to investigate the potential of a 1-year MedDiet intervention to bring changes in the gut microbiota and reduce frailty. The results of the study showed that adherence to the NU-AGE MedDiet led to specific alterations in the gut microbiome and was positively associated with markers of lower frailty and improved cognitive function while displaying negative associations with inflammatory markers like C-reactive protein and interleukin-17. The analysis of microbial metabolite profiles indicated that the changes in the microbiome due to the diet were linked to increased production of beneficial short/branch chained fatty acids and reduced production of secondary bile acids, p-cresols, ethanol, and carbon dioxide [78].

### 4.4. Ketogenic Diet

The fundamental principle of the ketogenic diet revolves around significantly restricting dietary carbohydrates while allowing for varying levels of protein and fat intake [79]. The traditional ketogenic diet is characterized by specific parameters, including a protein intake of one gram per kilogram of body weight, a daily carbohydrate limit of 10–15 g, and the allocation of the remaining caloric intake from fats [80]. Numerous studies have been conducted to elucidate the potential of the ketogenic diet in enhancing healthspan and lifespan in animal models. Long-term exposure to a ketogenic diet every other week from middle age reduced mid-life mortality and maintained cognitive function in aging male mice [81]. Also, the ketogenic diet (KD) extends lifespan and mitigates age-related decline in physiological function in adult mice, possibly through a cross-talk between histone deacetylase inhibition and liver mTORC1 signaling [82]. Evidence suggests that ketone bodies (KB) exert neuroprotective effects on aging brain cells, promoting enhanced mitochondrial function and reducing the expression of inflammatory and apoptotic mediators [83]. A ketogenic diet might be beneficial in the treatment of neurodegenerative disorders, such as Alzheimer’s disease [84] and Parkinson’s disease [85], especially since personalized treatment is highly recommended [86]. Although promising results are obtained in animal models, clinical trials mostly focus on KD effects in weight loss, with debating results on blood lipids and overall cardiovascular risk [87,88]. 

A single-site, randomized, crossover clinical trial involved 33 adults aged ≥18 years with prediabetes (HbA1c 5.7–6.4% or fasting glucose 100–125 mg/dL) or type 2 diabetes (HbA1c ≥ 6.5% or fasting glucose ≥ 126 mg/dL) and body mass index (BMI) < 40 kg/m^2^ compared two low-carb diets, the well-formulated ketogenic diet (WFKD) and the Mediterranean-plus diet (Med-Plus), for 12 weeks. Results revealed that HbA1c values did not significantly differ between the two diets. Triglyceride levels significantly decreased with the WFKD, and LDL cholesterol was higher with the WFKD. Weight decreased by 8% with the WFKD and 7% with the Med-Plus, showing a significant diet–order interaction. HDL cholesterol increased by 11% with the WFKD and 7% with the Med-Plus, also with a significant diet–order interaction. Participants in the WFKD had lower fiber and nutrient intakes [89].

In another controlled pilot trial involving 24 adults aged between 18 and 41 years with normal nutritional behavior and a body mass index of 18–27 kg/m^2^, participants in the intervention group followed a high-carbohydrate (HC) diet for 3 weeks, consisting of 75–80% carbohydrates, 15% proteins, and 5–10% fat. After a wash-out period of around 3 weeks, the second intervention phase began with a 1-week lead-in phase of a low-carbohydrate (LC) diet (20–25% carbohydrates, 15% proteins, 60–65% fat), followed by a 2-week ketogenic diet (5–7% carbohydrates, 15% proteins, 80% fat). The VO2 peak remained similar after both interventions, while the HC diet resulted in higher peak performance, longer time to exhaustion, and greater Watt/kg performance. Both diet arms saw significant reductions in body and fat mass compared to baseline, with no significant changes in lean body mass or skeletal muscle mass. The resting metabolic rate remained constant in both groups. The HC diet led to significant reductions in total cholesterol and LDL-cholesterol levels and increased triglycerides [90]. 

Nonetheless, the implementation of the ketogenic diet in the elderly population brings forth specific concerns, given that the KD often results in decreased appetite and potential gastrointestinal issues. It is imperative to conduct further research to thoroughly assess the appropriateness and safety of applying the ketogenic diet within this particular demographic [85]. The National Lipid Association Nutrition and Lifestyle Task Force states that limited evidence exists to support any distinct advantages of the ketogenic diet to impacts on cardiometabolic risk factors [91].

Table 1 outlines the primary outcomes associated with the dietary interventions in the reviewed clinical trials (Table 1). 

## 5. Discussion

Promoting healthy aging through dietary interventions that impact molecular pathways represents a promising frontier in gerontology and preventive medicine. Central to our review is the convergence of dietary interventions on key molecular pathways implicated in aging. By modulating nutrient-sensing pathways such as mTORC1, AMPK, and sirtuins, dietary regimens exert pleiotropic effects on cellular metabolism [92,93,94], stress resistance [95,96], and longevity [82,97]. 

The main effects of caloric restriction have been shown to facilitate weight loss, reduce inflammation, improve cardiometabolic health, and slow biological aging, making it a promising strategy for promoting healthy aging [58,59]. The effects on aging go primarily through modulation of the mechanistic target of the rapamycin signaling pathway. CR significantly impacts nutrient-sensing and signaling pathways critical to cellular metabolism and growth, notably reducing mTORC1 activity, which is highly sensitive to amino acid levels, particularly leucine. CR typically entails a decrease in protein intake, resulting in lower intracellular amino acid concentrations and diminished mTORC1 activation, thus influencing cellular growth processes [98]. Additionally, CR reduces insulin and IGF-1 signaling, major activators of mTORC1 via the PI3K/Akt pathway, due to decreased intake of carbohydrates and fats, leading to less phosphorylation and further reduced mTORC1 activity [99]. CR also activates AMP-activated protein kinase (AMPK) due to lower ATP levels, which then inhibits mTORC1 directly by phosphorylating Raptor and indirectly by activating the tuberous sclerosis complex (TSC1/2), a negative regulator of mTORC1 [100,101]. Furthermore, mTORC1 inhibition under CR conditions promotes autophagy, enhancing the breakdown and recycling of cellular components, which is crucial for cellular cleanup and homeostasis, particularly during nutrient scarcity [102,103].

Intermittent fasting demonstrates short-term improvements in body composition, cardiovascular markers, and cardiovascular risk scores, while also showing long-term benefits such as enhanced cholesterol levels, modulation of thyroid function, increased secretion of parathyroid hormone (PTH), lower levels of sICAM-1, and depletion of the pro-aging amino acid methionine [66,67]. While IF and CR share many common pathways related to aging and metabolism, the periodic nature of IF introduces variability in how these pathways are activated, potentially offering additional benefits or different impacts compared to continuous caloric restriction. Specifically, IF’s cyclic fasting and feeding periods may lead to more pronounced improvements in insulin sensitivity due to the extended periods without food intake, which helps regulate blood sugar levels dramatically [104,105]. This periodic metabolic stress may also enhance cellular stress response mechanisms more robustly than CR [106,107], upregulating protective pathways such as heat shock proteins and sirtuins, which contribute to cellular stress resilience and longevity.

The Mediterranean-like diet led to a significant decrease in BMI, with improvements in baseline epigenetic age acceleration and post-intervention measures. Furthermore, it was associated with significant enhancements in global cognition and episodic memory, along with specific microbiome alterations positively linked to markers of lower frailty, improved cognitive function, and reduced inflammatory markers such as C-reactive protein and interleukin-17 [76,77,78]. The Mediterranean diet similarly impacts these pathways but through nutrient-dense, high-quality foods rather than caloric or temporal restriction. For example, the high levels of unsaturated fats and antioxidants in the diet can modulate insulin sensitivity and endothelial function, indirectly affecting mTORC1 activity and promoting cardiovascular health. Additionally, the fiber-rich components of the diet support gut health and modulate the production of short-chain fatty acids, which have been shown to activate AMPK and potentially mimic some of the metabolic benefits seen with CR and IF [108,109].

The ketogenic diet is associated with significant reductions in triglyceride levels, increased HDL cholesterol, and substantial weight loss, albeit with higher LDL cholesterol levels. However, higher levels of LDL cholesterol were also identified, which could pose a potential risk for cardiovascular health in some individuals [89,90]. In the context of the reduction in glucose and insulin levels due to low carbohydrate intake directly decreases mTORC1 activity, typically stimulated by insulin and amino acids. This inhibition promotes autophagy and may help in cellular detoxification and longevity, akin to the mechanisms activated by CR and IF [110,111].

**Figure 2:**
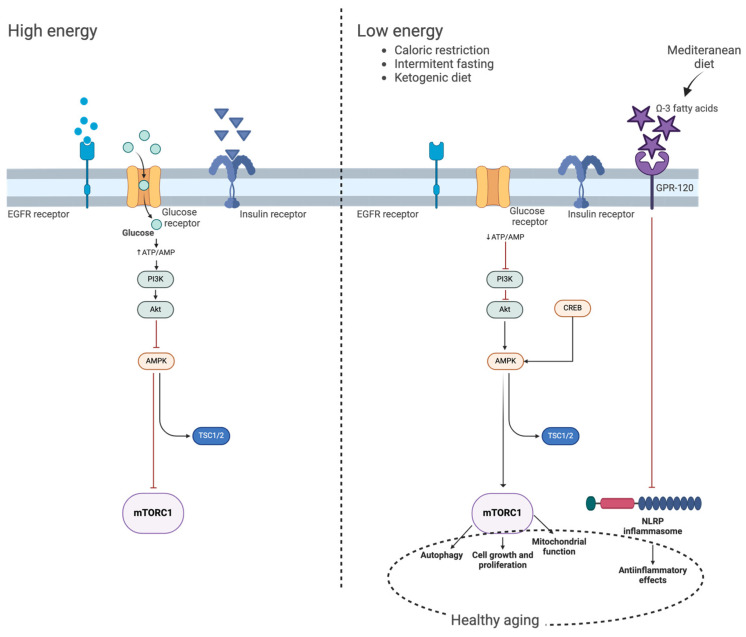
Main metabolic pathways and effect of caloric restriction, intermittent fasting, ketogenic diet, and Mediterranean diet (created with BioRender.com (accessed on 19 August 2024)).

Figure 2 serves as a visual summary of the main findings discussed in the text, encapsulating how various dietary interventions impact molecular pathways involved in healthy aging. It highlights the contrast between high-energy and low-energy states and their respective influences on critical signaling pathways like mTORC1, AMPK, and insulin signaling (Figure 2).

Despite the promise of dietary interventions in promoting healthy aging, several challenges warrant attention. Variability in study designs, participant characteristics, and dietary protocols complicates the interpretation and generalizability of findings. Addressing these challenges requires standardized methodologies, longitudinal studies, and interdisciplinary collaborations to elucidate causal relationships between diet, molecular pathways, and aging outcomes.

## 6. Strengths and Limitations

The strength of this study lies in its comprehensive consideration of the potential molecular pathways involved in the main dietary interventions for healthy aging. By examining evidence from microorganisms to animals and summarizing data from clinical trials, this approach provides a broad and beneficial contribution to the existing literature. However, there are several limitations to the study, including the specific timeframe covered, the English language filter applied, and the fact that it does not follow the rigorous approach of a systematic review.

## 7. Conclusions

In conclusion, dietary interventions hold significant potential in promoting healthy aging by influencing key molecular pathways involved in the aging process. While current research highlights the promising effects of these interventions, further evidence is needed to fully validate and refine personalized nutritional strategies for optimizing healthspan and mitigating age-related conditions. Moving forward, interdisciplinary efforts will be crucial to translating molecular insights into clinical practice and overcoming existing challenges in the application of dietary interventions to support healthy aging.

## 8. Future Directions

Future perspectives in research would be focused on chrononutrition in chronic diseases and the impact that intermittent fasting as well as different types of diet (Mediterranean, DASH) has on functional abilities and quality of life in older adults [112]. Another direction of research could be the redefinition of the tools used to quantify the diet-health aging relationship. We will see changes both in the use of diet assessment markers, which will replace the standardized questionnaires regarding eating habits currently used, as well as in how healthy aging is measured with the help of classical aging scores [112,113]. The studies will evaluate the long-term effects of both micronutrients and macronutrients on healthy aging across various age stages in order to develop comprehensive dietary recommendations [114]. Due to the significant socio-economic changes currently happening, the important phenomenon of population migration, different cultural contexts, and the influences of diet and behaviors during childhood and young adulthood are difficult to quantify [115]. 

## Figures and Tables

**Table 1:** Main effects of the dietary interventions and targeted markers.

Intervention	Targeting Markers	Cohort	Duration	Results	
Caloric restriction	Weight loss, T3 and TNF-α, lipidogram, RMR, TDEE	218 individuals, 21 to 51 yo	2 years	The intervention proved to be effective in facilitating weight loss, eliciting a decline in circulating TNF-α levels, and leading to a drop in cardiometabolic risk factors.	[58]
Caloric restriction	Klemera–Doubal method (KDM)	Follow-up (1)	2 years	During the CALERIE trial, caloric restriction led to slower biological aging compared to the ad libitum approach. Ad libitum participants saw a yearly increase in biological age of 0.71 years, while caloric restriction participants experienced a slower increase of 0.11 years per year, indicating notable deceleration of aging in the caloric restriction group.	[59]
Alternate day fasting	BMI, Framingham risk score, fT3, PTH, sICAM-1, methionine	60 individuals, 48 to 52 yo	4 weeks short term, >6 months long term	In the short term, ADF enhances body composition (reduction in BMI by over 1 kg/m^2^), cardiovascular markers, and reduces Framingham risk score of future adverse cardiovascular events by approximately 1.42%; in the long term, ADF reduced cardiovascular risk, improved cholesterol levels, and modulation of thyroid function, increased secretion of PTH and lowers levels of sICAM-1.	[66]
Time-restricted feeding	BMI, cardiovascular parameters, lipidogram, inflammation markers and plasma metabolites	24 healthy men and women, 55 to 79 yo	6 weeks	No significant impacts of TRF on cardiovascular health markers or cognitive performance were noted. The results imply that TRF could enhance functional capacity and glucose tolerance, as evidenced by improved 6 min walk distance, a reliable indicator of healthspan in older adults	[67]
Mediterranean diet	BMI, DNA methylation age and of epigenetic age acceleration	1279 participants, 65–79 yo	1 year	Italian males experienced a significant decrease in BMI (*p* = 0.008). Both Italian and Polish groups showed associations between baseline epigenetic age acceleration and post-intervention measures. The Mediterranean-like diet led to significant rejuvenation in Polish subjects based on the AgeAccel measure (−1.47 years of AgeAccelDiff and of −1.36 years of IEAADiff in the subgroup of Polish females)	[76]
Mediterranean diet	MMSE CERAD-total score	1279 adults, 65–79 yo	1 year	Higher adherence to the NU-AGE diet led to significant enhancements in global cognition (β = 0.20, *p* = 0.046) and episodic memory (β = 0.15, *p* = 0.025) compared to lower adherence individuals.	[77]
Mediterranean like diet NU-AGE	Microbiome profile, hsCRP, IL-17, sGP130, as well as adiponectin and leptin	612 participants, follow-up (6)	1 year	The study demonstrated that adherence to the NU-AGE MedDiet led to specific microbiome alterations and were positively linked to markers of lower frailty and improved cognitive function, while showing negative associations with inflammatory markers such as C-reactive protein and interleukin-17. Also, microbiome changes were associated with increased production of beneficial short/branch chained fatty acids and reduced production of secondary bile acids, p-cresols, ethanol, and carbon dioxide.	[78]
2 low-carb diets: WFKD and the Mediterranean-plus diet	HbA1c, fasting insulin and blood lipid values	33 adults with prediabetes or T2DM	12 weeks	HbA1c values showed no significant difference between the diets. Triglyceride levels decreased more significantly with the WFKD, while LDL cholesterol was higher for the WFKD. Weight decreased by 8% for the WFKD and 7% for the Med-Plus, HDL cholesterol increased by 11% for the WFKD and 7% for the Med-Plus.	[89]
High-carbohydrate diet for 3 weeks, one-week low-carbohydrate (LC) diet, followed by a 2-week ketogenic diet	VO2, LDL, Tg, fat and lean body mass	24 adults, 18–41 yo	3 weeks + 3 weeks	VO2peak was similar after both interventions. High-carbohydrate (HC) diets had higher peak performance, longer time to exhaustion, and higher Watt/kg performance. Body and fat mass decreased significantly in both diet arms compared to baseline, but lean body mass and skeletal muscle mass did not. Resting metabolic rate was constant in both groups. The HC diet significantly reduced total cholesterol and LDL-cholesterol while increasing triglycerides. In conclusion, a short-term high-carbohydrate (HC) diet improved peak performance, time to exhaustion, Watt/kg performance, and body composition. The HC diet reduced total and LDL-cholesterol but raised triglycerides.	[90]

WFKD—well-formulated ketogenic diet; RMR—resting metabolic rate; TDEE—total daily energy expenditure; MMSE—mini-mental state examination; CERAD—consortium to establish a registry for Alzheimer’s disease.

## Data Availability

The original contributions presented in the study are included in the review, further inquiries can be directed to the corresponding author.
